# Economic uncertainty and suicide in the United States

**DOI:** 10.1007/s10654-021-00770-4

**Published:** 2021-06-10

**Authors:** Sotiris Vandoros, Ichiro Kawachi

**Affiliations:** 1 King’s College London, Bush House, 30 Aldwych, London, WC2B 4BG UK; 2 Harvard T.H. Chan School of Public Health, Boston, MA USA

**Keywords:** Suicide, Economic uncertainty, Economic conditions, Unemployment, United States

## Abstract

Previous studies have found an association between recessions and increased rates of suicide. In the present study we widened the focus to examine the association between economic uncertainty and suicides. We used monthly suicide data from the US at the State level from 2000 to 2017 and combined them with the monthly economic uncertainty index. We followed a panel data econometric approach to study the association between economic uncertainty and suicide, controlling for unemployment and other indicators. Economic uncertainty is positively associated with suicide when controlling for unemployment [coeff: 8.026; 95% CI: 3.692–12.360] or for a wider range of economic and demographic characteristics [coeff: 7.478; 95% CI: 3.333–11.623]. An increase in the uncertainty index by one percent is associated with an additional 11–24.4 additional monthly suicides in the US. Economic uncertainty is likely to act as a trigger, which underlines the impulsive nature of some suicides. This highlights the importance of providing access to suicide prevention interventions (e.g. hotlines) during periods of economic uncertainty.

**Supplementary Information:**

The online version contains supplementary material available at 10.1007/s10654-021-00770-4.

## Introduction

There were over 48,000 suicides in the USA in 2018, demonstrating an upward trend since 2000 [1]. The literature has established a link between economic conditions and deteriorating mental health [2–5]. Suicides are generally associated with recessions and higher unemployment rates [6–12] as well as fiscal austerity [13, 14]. The effect often appears to be stronger for males than females [15, 16].

Monthly economic activity was shown to be inversely correlated with suicides in New York City, [17] but this did not hold for stock market volatility. By contrast, a recent study argues that there is a positive association between business performance and suicides, possibly due to stress put on young professionals [18]. Other studies found little evidence of a link between economic conditions and suicides [19, 20].

Apart from negative financial events that have already materialised (e.g. recessions and unemployment), we hypothesized that uncertainty about future events may also have a detrimental effect on mental health. Job insecurity or fear of job loss (a source of economic uncertainty) are associated with adverse health outcomes [21–24]. This affects the mental health of spouses as well, which is more evident in single-income households [25, 26].

Economic uncertainty can have an immediate impact on health outcomes. For example, there are more car crashes on days with higher levels of economic uncertainty, possibly due to distraction as a result of stress, anxiety and sleep deprivation [27]. Similarly, the announcement of austerity measures also led to temporary spikes in car crashes [28]. Car crashes have also been associated with stock market performance in the US [29].

When we turn to suicides in particular, daily economic uncertainty is associated with suicides in England and Wales [30]. Another study found a positive association between economic uncertainty and suicides in the USA, based on annual observations at the national level and using an ordinary least squares estimator, for the period 1950–2013 [31].

The objective of this paper is to study the association between monthly fluctuations in economic uncertainty and suicides in the United States from 2000 to 2017. Our study contributes to the literature by studying the impact of uncertainty rather than actual negative economic developments on health; by using data at the State level; by following a panel data approach on the link between economic uncertainty and suicide, thus controlling for unobserved heterogeneity across states; by using monthly data, consequently taking into account monthly fluctuations; and by providing recent empirical evidence from the world’s largest economy.

## Data and methods

We obtained data from the Center for Disease Prevention & Control (CDC) on the monthly number of suicides per State for the period from 2000 to 2017 [32]. Observations with fewer than 10 suicides per month were not reported by the data source, to prevent individuals from being identified. As a result, 1144 out of a total of 11,016 observations (from 13 out of 51 States) were dropped. We used the number of suicides per million inhabitants, to account for population differences across states. Population estimates were obtained from the Population Division of the U.S. Census Bureau [33].

To capture economic uncertainty, we used the monthly economic uncertainty index for the USA, obtained from “Economic Policy Uncertainty” [34, 35]. For robustness, we used three different indexes instead of relying on a single one: The three component index; the news-based index; and the economic policy uncertainty index. These indexes reflect the level of uncertainty by capturing the volume of terms associated with uncertainty in newspapers, and by measuring dispersion in economic forecasts and uncertainty surrounding future taxation.

First, the three component index includes three elements: News coverage, tax code expiration data, and economic forecaster disagreement. In particular, the news coverage element relies on the coverage of uncertainty by ten US newspapers (Boston Globe, Chicago Tribune, Dallas Morning News, Los Angeles Times, Miami Herald, New York Times, San Francisco Chronicle, USA Today, Wall Street Journal, Washington Post). Search results were used to capture the volume of economic policy uncertainty -related news reported in these newspapers. The search included a number of terms such as 'uncertain', 'federal reserve', 'deficit' etc [34]. The tax code expiration data took advantage of the fact that tax codes are often extended by Congress at the last minute, and was thus based on Congressional Budget Office reports, from which the authors obtained the tax codes that were due to expire in the next years. These were combined and weighted to capture the level of uncertainty relating to the direction that these taxes could take in the future [34]. The third element is based on the Survey of Professional Forecasters (conducted by the Federal Reserve of Philadelphia) which measures dispersion in forecasts of economic variables that are affected by monetary and fiscal policy [34]. The second way to measure uncertainty was to rely exclusively on the news-based index, derived from search results from ten major US newspapers, capturing terms relating to uncertainty [34]. The third approach employed the economic policy uncertainty index from the so-called Categorical Economic Policy Uncertainty Data. This index was constructed based on search results of keywords relating to economic uncertainty from more than 2,000 US newspapers in the Access World News database [34]. Further details on the indexes are available by the data source [34, 35].

Monthly unemployment rates by state for the period 2000–2017 were obtained from the Bureau of Labor Statistics [36]. Figure A1 in the online Appendix shows the trends of the uncertainty index, together with suicides and the unemployment rate, over the study period.

We also used additional data. Based on data from the US Census Bureau, [37] we identified whether a State reported a Budget surplus or deficit each year. We also obtained the State GDP growth rate between two subsequent periods [38] (available yearly from 2000 to 2004 and quarterly thereafter) and State poverty rates [39] (i.e. the percentage of people living in poverty, reported annually) from the Bureau of Economic Analysis. The average weekly unemployment benefit per person (reported quarterly) was obtained from the Employment & Training Administration of the US Department of Labor, [40] and was deflated using the monthly consumer price index obtained from the Bureau of Labor Statistics [41]. The percentage of people aged 19–65 in each State and the percentage of females as a proportion of the total population were calculated using annual data from the US Census Bureau [42, 43].

The analysis followed a panel data econometric approach. The panel identifier was the State, and suicides were reported monthly. When using panel data, the Fixed Effects estimator is always consistent, while the Random Effects estimator is more efficient, but not always consistent. We expect that the State-specific effects are correlated with the independent variables, which means that we should rely on the fixed effects estimator. This was confirmed by the Hausman test. We clustered standard errors at the State level in all regressions.

Our fixed effects model has the following general specification (Eq. 1):

1 $$suicides_{it} = \alpha_{i} + \beta \ln uncerta{\text{int}} y_{t} + \gamma X_{it} + \varepsilon_{it}$$

The dependent variable (*suicides*_*it*_) represents the number of suicides per million people in State *i* in month *t*. The main explanatory variable is ln*uncertainty* which captures the logarithm of the monthly economic uncertainty index. Vector *X*_it_ includes control variables: The monthly state unemployment rate in particular was included in the regression to filter out effects that might relate to actual economic conditions (such as a recession) rather than uncertainty per se. Depending on the specification, control variables may include the State GDP growth rate; a dummy variable for whether the state had a budget surplus or not; the poverty rate; the average weekly unemployment benefits; the percentage of the population aged between 18 and 65 capturing the part of the population that is more likely to be part of the workforce; and the percentage of the population that is female. We controlled for these variables due to the role that unemployment and financial conditions, [6–14] as well as poverty, [44] safety nets, [15, 45] age [46] and gender [15, 16] may play in suicides. We also included month—year dummies (as often used in the literature) [47–50] due to suicide seasonality and the fact that suicides have been demonstrating an upward yearly trend in the US. *ε*_*it*_ is the error term. Summary Statistics are presented in Table A1 in the Online Appendix.

## Results

Results of the baseline fixed effects panel data model (controlling for unemployment) are reported in Table 1. When considering the natural logarithm of the three-component uncertainty index as a determinant of suicide, the coefficient is positive and statistically significant [coeff: 8.026; 95% CI: 3.692–12.360], which means that a one percent increase in the index is associated with an additional 0.08 monthly suicides per million people. The unemployment rate does not appear to be associated with suicides [coeff: 0.010; 95% CI: − 0.105 to 0.125]. The magnitude of the effect appears to decrease when considering the natural logarithm of the news-based uncertainty index, but the association remains positive and statistically significant [coeff: 3.930; 95% CI: 1.808–6.052]. Again, the unemployment rate does not seem to be associated with suicides. Results are similar when using the economic policy uncertainty index [coeff: 4.745; 95% CI: 2.182—7.307], while the coefficient of unemployment remains insignificant.

**Table 1:** Baseline fixed effects regression results

	(1)	(2)	(3)
Natural logarithm of the three-component index	8.026*** [3.692 to 12.360]		
Natural logarithm of the news-based index		3.930*** [1.808 to 6.052]	
Natural logarithm of the uncertainty index			4.745*** [2.182 to 7.307]
unemployment rate	0.010 [− 0.105 to 0.125]	0.010 [− 0.105 to 0.125]	0.010 [− 0.105 to 0.125]
Constant term	− 24.961** [− 44.950 to -4.972]	− 6.557 [− 16.616 to 3.503]	− 8.101 [− 18.993 to 2.791]
R-squared within	0.288	0.288	0.288

The dependent variable is the number of suicides per million people (*suicides*). Fixed effects at the State level. Month-year dummies are used as controls in the regressions. Confidence intervals in brackets. Standard errors are clustered at the State level. *N* = 9872

***p < 0.01, **p < 0.05, *p < 0.1

To further validate that results are actually measuring uncertainty and not the consequences of a recession, we examine whether findings are robust to the inclusion of lagged values of unemployment rates. Table 2 presents results of a specification that includes the first lag of the unemployment rate as a control. Results are close to those of the baseline model and hold the same interpretation. The coefficient of the natural logarithm of uncertainty is positive and statistically significant in all cases, whether considering the three-component index [coeff: 9.026; 95% CI: 7.151–10.901], the news-based index [coeff: 5.043; 95% CI: 3.995–6.090], or the economic policy uncertainty index [coeff: 6.437; 95% CI: 5.100–7.774]. With regards to the lagged unemployment variable, the coefficient is statistically insignificant in all three cases. As a robustness check, we ran the same regressions without including any indicator of unemployment. Results (reported in Table 3) were very similar to that of the baseline model and hold the same interpretation.

**Table 2:** Results of the fixed effects regression with lagged unemployment rates

	(1)	(2)	(3)
Natural logarithm of the three component index	9.026*** [7.151 to 10.901]		
Natural logarithm of the news based index		5.043*** [3.995 to 6.090]	
Natural logarithm of the uncertainty index			6.437*** [5.100 to 7.774]
Lag of unemployment rate	0.017 [− 0.094 to 0.128]	0.017 [− 0.094 to 0.128]	0.017 [− 0.094 to 0.128]
Constant term	− 29.700*** [− 38.059 to -21.341]	− 12.083*** [− 16.796 to -7.370]	− 15.605*** [− 21.045 to -10.165]
R-squared within	0.297	0.297	0.297

The dependent variable is the number of suicides per million people (*suicides*). Fixed effects at the State level. Month-year dummies are used as controls in the regressions. Confidence intervals in brackets. Standard errors are clustered at the State level. *N* = 9053

***p < 0.01, **p < 0.05, *p < 0.1

**Table 3:** Fixed effects regression results without controlling for unemployment

	(1)	(2)	(3)
Natural logarithm of the three component index	8.032*** [3.730 to 12.334]		
Natural logarithm of the news based index		3.933*** [1.826 to 6.039]	
Natural logarithm of the uncertainty index			4.748*** [2.205 to 7.292]
Constant term	− 24.950** [− 44.991 to -4.909]	− 6.532 [− 16.713 to 3.649]	− 8.077 [− 19.084 to 2.931]
R-squared within	0.288	0.288	0.288

The dependent variable is the number of suicides per million people (*suicides*). Fixed effects at the State level apply. Month-year dummies are used as controls in the regressions. Confidence intervals in brackets. Standard errors are clustered at the State level. *N* = 9872

***p < 0.01, **p < 0.05, *p < 0.1

We also studied whether results are robust to the inclusion of a wider range of covariates, by controlling for the GDP growth rate; the presence of a budget surplus; poverty rates; the average weekly unemployment benefit; age; and gender composition (Table A2 in the Online Appendix). Once again, uncertainty is positively associated with suicides in all three models [Three-component index coeff: 7.478; 95% CI: 3.333–11.623; news-based index coeff: 3.662; 95% CI: 1.632–5.691; economic policy uncertainty index coeff: 4.421; 95% CI: 1.971–6.871]. We performed sensitivity analyses by running regressions with these additional economic indicators but without demographics; and by including demographics only. Results are reported in Tables A3 and A4, respectively, and once again confirm the results of the baseline model.

We also checked whether results are robust to using the uncertainty indexes in levels instead of logarithmic form (results reported in Table A5 in the Online Appendix). The positive relationship between all three indexes capturing economic uncertainty and suicide is once again confirmed. Finally, is unemployment associated with suicides, when removing uncertainty from the model? Results suggest a statistically insignificant association between unemployment and suicide (Table A6 in the Online Appendix).

## Discussion

We examined the link between economic uncertainty and suicide in the United States and found a positive association. An increase in the economic policy uncertainty index by one percent is associated with an increase in the monthly number of suicides by between 11 and 24.4, depending on the specification and uncertainty index used, which is equivalent to a 0.33–0.72% increase.

Our findings add to those of previous studies that suggest that economic uncertainty is associated with suicides in the UK [30] and the US. [31] They also add to a growing body of literature on the link between the short-term effects of uncertainty, bad financial news and events at the national level on health [17, 28, 29, 51–53].

We did not find any association between unemployment and suicide, which may initially sound surprising, as a number of studies have suggested that recessions are associated with increased suicide rates [6–12]. Nevertheless, others have found a negative association, suggesting that suicides can sometimes increase in a booming economy, [18, 54] or a weak or inexistent association between economic activity and suicide rates [19, 20].

People who are not unemployed might still face increased levels of economic uncertainty. For example, during the 2008 Great Recession, people may not have lost their jobs, but they might have been greatly affected by the crash in housing prices, resulting in them “going underwater”, i.e., owing more on their mortgages than the value of their houses. A big increase in indebtedness is likely to have led to financial worries, and may have affected planning for retirement, etc.

It is important to note that while high levels of uncertainty can occur at times of economic crises, these do not always occur simultaneously. For example, there was an increase in economic uncertainty in the US following the 2016 Presidential election, while unemployment was steadily decreasing (Figure A1 in the online Appendix). In addition, while the UK demonstrated record low unemployment rates between 2016 and 2019, economic uncertainty had reached very high levels following the Brexit referendum. The correlation between uncertainty and unemployment at the state level is 0.34, and Figure A1 shows that the uncertainty index is much more volatile than the unemployment rate, and trends are not always the same.

This study is subject to limitations. Observations with fewer than 10 suicides are not reported by the data source and were thus excluded from the sample. Furthermore, the unit of observation was monthly suicides, so there was no information on individual circumstances that may be associated with this outcome.

Economic uncertainty alone is unlikely to be the sole cause of suicide, but it may contribute to this phenomenon and act as a trigger for some individuals. This also highlights the role of impulsivity in some suicides [55–57]. Launching a three-digit suicide hotline (which is more memorable than a longer regular number) swiftly, instead of waiting for the scheduled launch of July 2022, is urgent, especially in light of the consequences of the Covid-19 pandemic and the associated financial consequences [58]. Our results also indicate that the timing of preventive measures, such as raising awareness, is of particular importance, and relevant information campaigns may need to intensify in periods of increased uncertainty.

Economic uncertainty reached unprecedented levels in the US in 2020 during the Covid-19 pandemic, and the index has also increased dramatically globally [34]. About half of the forecasted output contraction reflects a negative effect of COVID-induced uncertainty [59]. Future research can study the impact of uncertainty on suicides in this turbulent period.

## Supplementary Information

Below is the link to the electronic supplementary material.

Supplementary file1 (PDF 408 kb)
