# Supplementary material for: Economic uncertainty and suicide in the United States
Source: Eur J Epidemiol. 2021 Jun 10;36(6):641–7. doi: 10.1007/s10654-021-00770-4 (PMC8191707; doi:10.1007/s10654-021-00770-4)
Supplement: Supplementary file 1 — Supplementary file1 (PDF 408 kb) [file 10654_2021_770_MOESM1_ESM.pdf]

ONLINE APPENDIX

Table A1 – Summary Statistics

Variable	Mean	Std. Dev.	Min	Max
Monthly suicides per million people (state-level)	11.81	4.10	3.27	40.55
Three component index	113.70	35.99	57.20	245.13
News-based index	120.71	45.98	44.78	283.67
Uncertainty index	95.80	42.87	37.27	234.09
Unemployment rate	5.72	2.00	2.10	14.60
State GDP growth rate (compared to previous period)	1.90	4.78	-25.10	58.10
State budget surplus dummy	0.70	0.46	0	1
State poverty rate	12.70	3.42	4.50	25.80
Average weekly benefit per capita	133.11	24.22	81.80	244.72
Percentage of population female	50.78	0.77	47.92	52.93
Percentage of population aged 19 - 65	61.94	1.68	57.77	70.95

Table A2 – Results of the fixed effects regression with additional control variables

	(1)	(2)	(3)
Natural logarithm of the three component index	7.478***		
	[3.333 - 11.623]		
Natural logarithm of the news based index		3.662***	
		[1.632 - 5.691]	
Natural logarithm of the uncertainty index			4.421***
			[1.971 - 6.871]
unemployment rate	0.012	0.012	0.012
	[-0.084 - 0.107]	[-0.084 - 0.107]	[-0.084 - 0.107]
Constant term	65.623**	82.771***	81.332***
	[3.013 - 128.233]	[23.232 - 142.310]	[21.587 - 141.077]
R-squared within	0.292	0.292	0.292

The dependent variable is the number of suicides per million people (*suicides*). Additional control variables include the GDP growth rate; a dummy variable for budget surplus; the poverty rate; the average weekly benefit; the percentage of the population that is female; and the percentage aged 19-65. Fixed effects at the State level. Month-year dummies are used as controls in the regressions. Confidence intervals in brackets. Standard errors are clustered at the State level. *N*=9872. *** p<0.01, ** p<0.05, * p<0.1.

Table A3 – Sensitivity analysis A (excluding demographic variables)

	(1)	(2)	(3)
Natural logarithm of the three component index	7.944***		
	[3.737 - 12.151]		
Natural logarithm of the news based index		3.890***	
		[1.830 - 5.950]	
Natural logarithm of the uncertainty index			4.696***
			[2.209 - 7.183]
unemployment rate	0.012	0.012	0.012
	[-0.103 - 0.128]	[-0.103 - 0.128]	[-0.103 - 0.128]
Constant term	-25.152**	-6.937	-8.465
	[-44.578 - -5.727]	[-16.815 - 2.941]	[-19.137 - 2.207]
R-squared within	0.2882	0.2882	0.2882

The dependent variable is the number of suicides per million people (*suicides*). Additional control variables include the GDP growth rate; a dummy variable for budget surplus; the poverty rate; and the average weekly benefit. Fixed effects at the State level apply. Month-year dummies are used as controls in the regressions. Confidence intervals in brackets. Standard errors are clustered at the State level. *N*=9872. *** p<0.01, ** p<0.05, * p<0.1.

Table A4 – Sensitivity Analysis B (demographic controls only)

	(1)	(2)	(3)
Natural logarithm of the three component index	7.458***		
	[3.211 - 11.704]		
Natural logarithm of the news based index		3.652***	
		[1.572 - 5.731]	
Natural logarithm of the uncertainty index			4.409***
			[1.899 - 6.919]
unemployment rate	0.007	0.007	0.007
	[-0.090 - 0.104]	[-0.090 - 0.104]	[-0.090 - 0.104]
Constant term	63.832*	80.933**	79.498**
	[-5.748 - 133.411]	[14.186 - 147.680]	[12.564 - 146.433]
R-squared within	0.2917	0.2917	0.2917

The dependent variable is the number of suicides per million people (*suicides*). Additional control variables include the percentage of the population that is female; and the percentage of the population aged 19-65. Fixed effects at the State level apply. Month-year dummies are used as controls in the regressions. Confidence intervals in brackets. Standard errors are clustered at the State level. *N*=9872. *** p<0.01, ** p<0.05, * p<0.1.

Table A5 – Fixed Effects Regression results using uncertainty measured in levels

	(1)	(2)	(3)
three component index	0.080***		
	[0.037 - 0.124]		
news based index		0.035***	
		[0.016 - 0.054]	
uncertainty index			0.069***
			[0.032 - 0.106]
unemployment rate	0.010	0.010	0.010
	[-0.105 - 0.125]	[-0.105 - 0.125]	[-0.105 - 0.125]
Constant	3.919*	7.980***	7.177***
	[-0.507 - 8.345]	[5.706 - 10.254]	[4.483 - 9.871]
Observations	9,872	9,872	9,872
R-squared within	0.288	0.288	0.288

The dependent variable is the number of suicides per million people (*suicides*). Fixed effects at the State level apply. Month-year dummies are used as controls in the regressions. Confidence intervals in brackets. Standard errors are clustered at the State level. *N*=9872. *** p<0.01, ** p<0.05, * p<0.1.

Table A6 – Fixed Effects Regression results: The relationship between unemployment and suicide, without controlling for uncertainty

unemployment rate	0.010
	[-0.105 - 0.125]
year month dummies	yes
Constant	11.083***
	[10.283 - 11.882]
Observations	9,872
R-squared within	0.288

The dependent variable is the number of suicides per million people (*suicides*). Fixed effects at the State level apply. Month-year dummies are used as controls in the regressions. Confidence intervals in brackets. Standard errors are clustered at the State level. *N*=9872. *** p<0.01, ** p<0.05, * p<0.1.

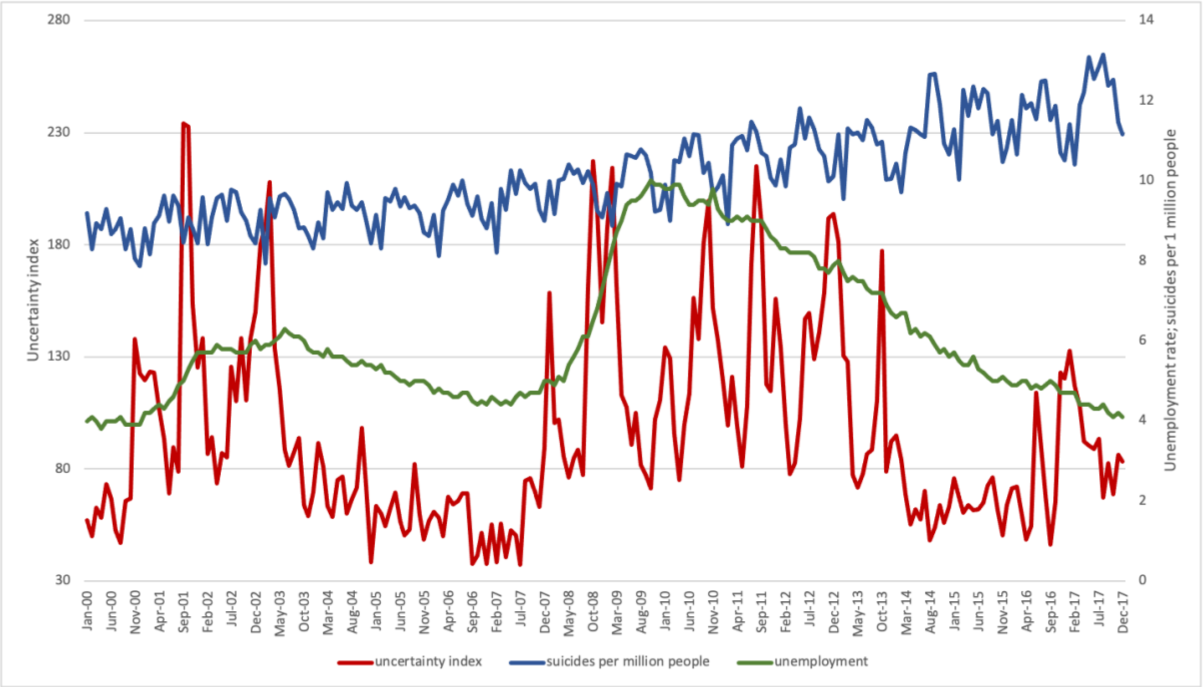

Figure A1 – Monthly suicides per million people, economic uncertainty index and unemployment rate in the US, from 2000 to 2017
